# The regio-selective synthesis of 10-hydroxy camptothecin norcantharidin conjugates and their biological activity evaluation *in vitro*

**DOI:** 10.1098/rsos.172317

**Published:** 2018-06-13

**Authors:** Chang K. Zhao, Chan Li, Xian H. Wang, Yu J. Bao, Fu H. Yang, Mei Huang

**Affiliations:** 1 School of Pharmacy, Zunyi Medical University, No. 6 Xue Fu West Road, Xin Pu New District, Zunyi City, 563003, Guizhou Province, People's Republic of China; 2 School of Pharmacy, Sun Yat-Sen University, 135 Xin Gangxi Road, Haizhu District, Guangzhou 563003, Guangdong Province, People's Republic of China

**Keywords:** 10-hydroxy camptothecin, pro-drug, regio-selective, conjugate, *in vitro*

## Abstract

A series of conjugates of 10-hydroxy camptothecin (HCPT) with functionalized norcantharidin derivatives were regio-selectively synthesized in the condition of (3-dimethylaminopropyl) ethyl-carbodiimide monohydrochloride in a moderate yield. The synthesized conjugate HCPT pro-drugs can also suppress cancer cell growth *in vitro*. These conjugated pro-drug constructs possess therapeutic potential as novel bi-functional conjugate platforms for cancer treatment.

## 1. Introduction

Topoisomerase I, a vital DNA-manipulating enzyme, is the only known target for camptothecin [1–3], which binds to the interface of the covalent protein–nucleic acid complex. 20-(*S*)-Camptothecin (CPT) was discovered in the early 1960s, by Wani's group at the NCI, from an extract of the bark of the Chinese tree *Camptotheca acuminata*—a common deciduous tree used for ages in traditional Chinese medicine. Owing to the high toxicity and low bioavailability, camptothecin was not usable as an anticancer agent *in vivo* [4,5].

**Figure 1.**
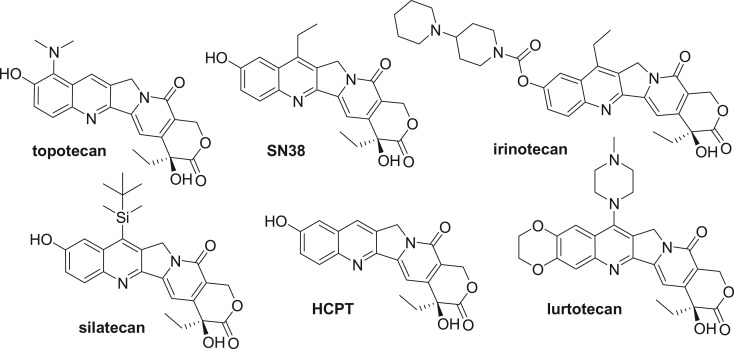
Structure of HCPT and derivatives.

It was discovered that the quinoline moiety of CPT could be substituted without loss of activity. This bicyclic moiety can be modified with a large amount of functional groups [6], especially the introduction of hydroxyl group in 10-position, while preserving and even in some cases promoting the cytotoxic action of the parent natural product. Structure reactivity relationship studies of camptothecin derivatives summarized that a hydroxyl substitution at the 10-position, such as topotecan and irinotecan, enhanced antitumour activity [7]. This led to the design and development of the two major anticancer drugs topotecan and irinotecan [8] (figure 1).

In general, for a given cancer there are several operating cancer genes or pathways. At the same time, metastasis is a major obstacle to anticancer therapies and responsible for most therapeutic failures, while drug resistance is always going to be a concern. Given these things, the dual target drug will be needed for optimal therapeutic effect [9–13].

In our ongoing project, 10-hydroxy camptothecin (HCPT) was chosen as a main anticancer pro-drug to conjugate with another anticancer drug norcantharidin owing to its unique feature of stimulation of the bone marrow production of white cells, which is in contrast to most other anticancer drugs that readily induce myelo-suppression [14–16]. The dual anticancer drug assemblies [17,18] were thus constructed, because HCPT is a DNA-topoisomerase I inhibitor and norcantharidin will also suppress cancer cell growth by inhibiting protein phosphatase.

**Figure 2.**
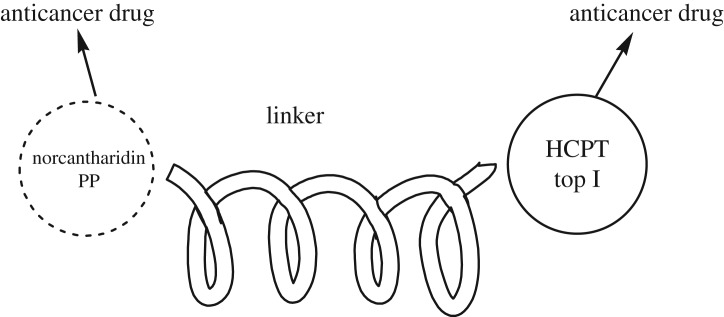
Structure of conjugate HCPT with other anticancer drugs.

With the encouragement of irinotecan and other similar derivatives, we supposed that a conjugate of HCPT with norcantharidin in 10-phenolic ester may improve its efficiency (figure 2). The research result is summarized in the following.

## 2. Material and methods

^1^H NMR spectra were recorded at 400 MHz on a Varian Unity INOVA 400 MHz NMR spectrometer using tetramethylsilane as an internal standard, and ^13^C NMR spectra were recorded at 100 MHz. Mass spectra were run on a Waters UPLC-MS instrument. Melting points were determined by a Mettler Toledo FP62 melting point apparatus and are uncorrected. Thin-layer chromatography plates (GF 254) were bought from Branch Qingdao Haiyang Chemical Plant.

## 3. Experimental

### 3.1. Chemistry

1. *Synthesis of side chain* 2

**Scheme 1.**
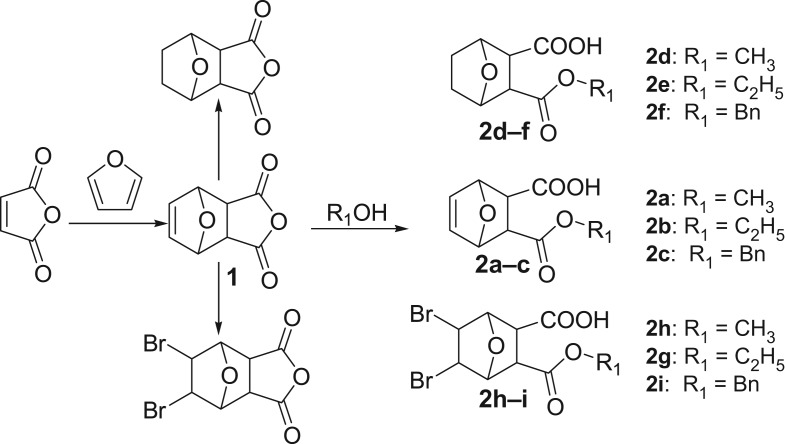
Synthesis of side chains **2a–i**.

The side chains **2a–i** were easily prepared by the following methods according to the literature (scheme 1).

**Scheme 2.**
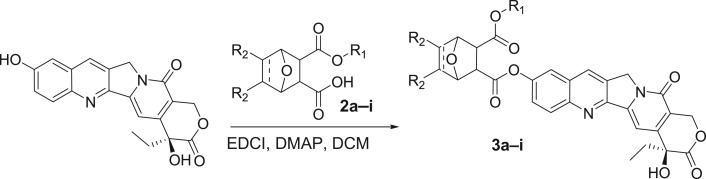
Synthesis of target compounds **3a–i**.

2. *Regio-selective synthesis of compounds* ***3a–i*** (*scheme 2*)

3. *A typical synthesis procedure for compounds* ***3a–i***

HCPT (100 mg, 0.27 mmol), 7-oxabicyclo[2.2.1]hept-5-en-2,3-dicarboxylic acid monomethyl ester (**2a**, 107 mg, 0.54 mmol), (3-dimethylaminopropyl)ethyl-carbodiimide monohydrochloride (EDCI) (124.0 mg, 1.29 mmol) and 4-dimethylaminopyridine (DMAP) (27.40 mg, 0.17 mmol) were suspended in CH_2_Cl_2_ (15 ml) and stirred at room temperature for 48 h. CH_2_Cl_2_ (40 ml) was added to dilute the reaction mixture. The mixture was then washed with H_2_O (20 ml × 3). The combined organic layers were dried over MgSO_4_. The solvent was removed under reduced pressure, and the residue was purified by flash column chromatography, eluting with CH_2_Cl_2_:CH_3_OH (= 97:3) to afford the title compound (**3a**, 103 mg, 69%) as a yellow solid. *R_f_* = 0.57 (CH_2_Cl_2_: CH_3_OH = 10: 1); mp: 149.6–150.2. ^1^H NMR (400 MHz, CDCl_3_) *δ* = 8.30 (s, 1H), 8.19 (d, *J* = 9 Hz, 1H), 7.72 (s, 1H), 7.65 (s, 1H), 7.60 (d, *J* = 9 Hz, 1H), 6.56 (s, 2H), 5.72 (d, *J* = 12 Hz, 1H), 5.50 (s, 1H), 5.32 (d, *J* = 16 Hz, 2H), 5.25 (s, 2H), 4.03 (s, 1H), 3.75 (s, 3H), 3.08 (q, *J* = 8 Hz, 2H), 1.87–1.92 (m, 2H), 1.02 (t, *J* = 8 Hz, 3H). ^13^C NMR (100 MHz, CDCl_3_) *δ* = 173.82, 171.76, 170.09, 157.55, 152.33, 150.08, 149.57, 146.79, 146.11, 136.84, 136.69, 131.15, 130.80, 129.03, 128.39, 125.70, 118.78, 118.67, 98.12, 81.10, 80.48, 72.77, 66.28, 52.65, 50.00, 47.48, 46.92, 31.60, 7.84. IR (KBr): *ν* (cm^−1^) = 3441, 3127, 2977, 1747, 1660, 1606, 1557, 1502, 1436, 1396, 1231, 1191, 1149, 1046, 913, 836, 723, 593.

Compound **3b** (65%); a yellow solid; mp: 159.2–160.8; *R_f_* = 0.55 (CH_2_Cl_2_ : CH_3_OH = 20 : 1); mp: 136.1–138.8. ^1^H NMR (400 MHz, CDCl_3_) *δ* = 8.32 (s, 1H), 8.21 (s, 1H), 8.19 (s, 1H), 7.76 (d, *J *= 2.4 Hz, 1H), 7.62–7.67 (m, 2H), 6.56 (s, 2H), 5.73 (d, *J *= 16 Hz, 1H), 5.51 (s, 1H), 5.27–5.33 (m, 4H), 4.19–4.23 (m, 2H), 3.92 (s, 1H), 3.07 (q, *J* = 8 Hz, 2H), 1.85–1.91 (m, 2H), 1.25 (t, *J* = 8 Hz, 3H), 1.03 (t, *J* = 8 Hz, 3H). ^13^C NMR (100 MHz, CDCl_3_) *δ* = 173.88, 171.28, 170.13, 157.57, 152.23, 150.08, 149.64, 146.84, 146.19, 136.87, 136.67, 131.15, 130.79, 129.00, 128.43, 125.76, 118.74, 118.69, 98.07, 81.24, 80.42, 72.75, 65.31, 61.58, 50.01, 47.59, 45.85, 31.59, 14.17, 7.84. IR (KBr): *ν* (cm^−1^) = 3423, 3097, 2975, 2928, 1750, 1660, 1606, 1557, 1503, 1484, 1367, 1231, 1181, 1148, 1047, 915, 815, 722, 595.

Compound **3c** (44%); a yellow solid; *R_f_* = 0.48 (CH_2_Cl_2_: CH_3_OH = 10: 1); mp: 135.5–137.2. ^1^H NMR (400 MHz, DMSO-d_6_) *δ* = 8.65 (s, 1H), 8.17 (d, *J *= 8 Hz, 1H), 7.74 (s, 1H), 7.51 (d, *J *= 8 Hz, 1H), 7.29–7.34 (m, 6H), 6.55 (s, *2*H), 5.41 (s, 2H), 5.37 (s, 1H), 5.28 (s, 2H), 5.24 (s, 1H), 5.14 (d, *J* = 8 Hz, 2H), 3.16 (q, *J* = 8 Hz, 2H), 1.82–1.89 (m, 2H), 0.87 (t, *J* = 8 Hz, 3H). ^13^C NMR (100 MHz, DMSO-d_6_) *δ* = 172.92, 171.63, 170.68, 157.23, 153.01, 150.44, 149.34, 146.29, 145.78, 137.29, 137.14, 136.28, 131.71, 131.70, 130.86, 128.93, 128.92, 128.86, 128.67, 128.53, 128.46, 126.16, 119.57, 119.39, 97.16, 80.95, 80.47, 72.82, 66.74, 65.68, 50.70, 47.27, 46.75, 30.69, 8.24. IR (KBr): *ν* (cm^−1^) = 3397, 3132, 3022, 2964, 1756, 1658, 1600, 1557, 1506, 1400, 1360, 1282, 1234, 1180, 1140, 1060, 910, 847, 811, 747, 477.

Compound **3d** (67.3%); a yellow solid; *R_f_* = 0.6 (CH_2_Cl_2_ : CH_3_OH = 10 : 1); mp: 154.3–155.7. ^1^H NMR (400 MHz, CDCl_3_) *δ* = 8.24 (s, 1H), 8.15 (d, *J* = 8 Hz, 1H), 7.66 (d, *J *= 16 Hz, 2H), 7.53 (d, *J *= 8 Hz, 1H), 5.69 (d, *J* = 16 Hz,1H), 5.14–5.28 (m, 4H), 4.96 (s, 1H), 4.38 (s, 1H), 3.72 (s, 3H), 3.25 (s, 2H), 1.83–1.90 (m, 4H), 1.64 (d, *J *= 8 Hz, 2H), 0.97 (t, *J* = 8 Hz, 3H). ^13^C NMR (100 MHz, CDCl_3_) *δ* = 173.75, 171.47, 169.59, 157.51, 152.23, 150.04, 149.48, 146.57, 146.94, 131.00, 130.83, 129.03, 128.30, 125.66, 118.83, 118.67, 118.65, 98.27, 79.07, 78.40, 72.85, 66.19, 52.74, 52.48, 51.78, 49.98, 31.59, 29.06, 7.84. IR (KBr): *ν* (cm^−1^) = 3443, 3130, 2988, 1746, 1660, 1606, 1503, 1435, 1399, 1231, 1191, 1142, 1054, 999, 817, 555.

Compound **3e** (66.3%); a white solid; *R_f_* = 0.33 (CH_2_Cl_2_ : CH_3_OH = 20 : 1); mp: 157.1–159.7. ^1^H NMR (400 MHz, DMSO-d_6_) *δ* = 8.65 (s, 1H), 8.18 (d, *J *= 8 Hz, 1H), 7.78 (s, 1H), 7.58 (d, *J *= 8 Hz, 1H), 7.31 (s, 1H), 6.52 (s, 1H), 5.40 (s, 2H), 5.24 (s, 2H), 4.95 (s, 1H), 4.79 (s, 1H), 4.07 (d, *J *= 8 Hz, 2H), 3.41 (d, *J *= 8 Hz, 2H), 1.85 (s, 2H), 1.64 (s, 4H), 1.13 (s, 3H), 0.87 (s, 3H). ^13^C NMR (100 MHz, DMSO-d_6_) *δ* = 172.89, 171.36, 170.17, 157.19, 152.93, 150.42, 149.35, 146.26, 145.74, 131.65, 130.85, 130.79, 128.67, 126.12, 119.54, 119.32, 97.20, 78.74, 78.23, 72.82, 65.68, 60.93, 52.29, 51.31, 50.63, 30.74, 28.92, 14.47, 8.22. IR (KBr): *ν* (cm^−1^) = 3474, 3414, 3130, 2984, 1745, 1659, 1615, 1502, 1399, 1231, 1191, 1144, 1051, 998.

Compound **3f** (44.6%); a yellow solid; *R_f_* = 0.58 (CH_2_Cl_2_ : CH_3_OH = 20 : 1); mp: 245.2–248.1. ^1^H NMR (400 MHz, DMSO-d_6_) *δ* = 8.62 (s, 1H), 8.15 (d, *J *= 8 Hz, 1H), 7.71 (s, 1H), 7.48 (d, *J *= 12 Hz, 1H), 7.31 (d, *J *= 12 Hz, 6H), 6.55 (s, 1H), 5.41(s, 2H), 5.26 (s, 2H), 5.11 (q, *J *= 12 Hz, 2H), 4.97 (s, 1H), 4.83 (s, 1H), 3.44 (d, *J *= 4 Hz, 2H), 1.86 (t, *J *= 16 Hz, 2H), 1.65 (s, 4H), 0.87 (s, 3H). ^13^C NMR (100 MHz, DMSO-d_6_) *δ* = 172.93, 171.28, 170.18, 160.50, 152.97, 152.93, 150.42, 149.29, 146.25, 136.31, 131.65, 130.80, 128.86, 128.51, 128.45, 126.15, 122.43, 119.53, 119.39, 97.14, 88.06, 86.28, 78.83, 78.81, 78.27, 72.82, 66.54, 65.69, 55.00, 52.24, 51.29, 50.67, 30.68, 28.92, 8.23. IR (KBr): *ν* (cm^−1^) = 3419, 3130, 2975, 1757, 1739, 1660, 1558, 1504, 1456, 1400, 1360, 1298, 1233, 1181, 1145, 1062, 1001, 839, 741.

Compound **3g** (62.2%); a yellow solid; *R_f_* = 0.33 (CH_2_Cl_2_: CH_3_OH = 97: 3), mp: 181.9–183.3. ^1^H NMR (400 MHz, DMSO-d_6_) *δ* = 8.65 (s, 1H), 8.17 (d, *J *= 8 Hz, 1H), 7.89 (d, *J *= 8 Hz, 1H), 7.60–7.73 (m, 1H), 7.30 (s, 1H), 6.53 (s, 1H), 5.22 (s, 2H), 5.11 (s, 1H), 4.96 (t, *J *= 8 Hz, 1H), 4.45–4.67 (m, 2H), 3.98–4.03 (m, 1H), 3.83–3.87 (m, 1H), 3.66–3.75 (m, 3H), 1.85 (t, *J *= 4 Hz, 2H), 0.87 (t, *J *= 8 Hz, 3H). ^13^C NMR (100 MHz, DMSO-d_6_) *δ* = 172.89, 171.86, 170.19, 169.28, 168.35, 157.15, 153.03, 150.38, 149.04, 146.35, 145.66, 131.70, 130.95, 128.61, 125.93, 119.58, 97.19, 86.84, 85.60, 82.34, 72.80, 65.68, 54.48, 52.85, 50.59, 49.88, 46.26, 30.73, 8.24. IR (KBr): *ν* (cm^−1^) = 3473, 3415, 3161, 1744, 1657, 1611, 1400, 1152, 617, 479.

Compound **3h** (55.3%); a yellow solid; *R_f_* = 0.34 (CH_2_Cl_2_ : CH_3_OH = 20 : 1); mp: 167.8–169.3. ^1^H NMR (400 MHz, DMSO-d_6_) *δ* = 8.64 (s, 1H), 8.16 (d, *J *= 8 Hz, 1H), 7.82–7.94 (m, 1H), 7.59–7.69 (m, 1H), 7.29 (s, 1H), 6.52 (s, 1H), 5.39 (s, 2H), 5.09–5.26 (m, 3H), 4.92–4.98 (m, 1H), 4.45–4.68 (m, 2H), 4.10–4.21 (m, 2H), 3.97–4.03 (m, 1H), 3.67–3.82 (m, 1H), 1.81–1.88 (m, 2H), 1.22–1.26 (m, 1H), 1.15 (t, *J *= 8 Hz, 2H), 0.87 (t, *J *= 8 Hz, 3H). ^13^C NMR (100 MHz, DMSO-d_6_) *δ* = 172.88, 169.70, 169.20, 168.40, 157.14, 153.01, 150.39, 149.05, 146.32, 145.65, 131.68, 130.79, 128.58, 125.89, 119.56, 97.19, 86.90, 82.33, 72.80, 65.68, 62.09, 61.65, 54.53, 50.60, 49.96, 46.20, 30.74, 14.39, 8.24. IR (KBr): *ν* (cm^−1^) = 3474, 3414, 3232, 3146, 1744, 1658, 1616, 1502, 1399, 1230, 1153, 636, 479.

Compound **3i** (48%); a yellow solid; *R_f_* = 0.33 (CH_2_Cl_2_ : CH_3_OH = 10 : 1); mp: 147.2–147.6. ^1^H NMR (400 MHz, CDCl_3_) *δ* = 8.24 (s, 1H), 8.14 (d, *J *= 8 Hz, 1H), 7.62–7.64 (m, 2H), 7.46 (d, *J *= 8 Hz, 1H), 7.31–7.34 (m, 5H), 7.24 (s, 1H), 5.70 (d, *J *= 16 Hz, 1H), 5.22 (d, *J *= 8 Hz, 1H), 5.14 (s, 1H), 5.08–5.10 (m, 1H), 5.02 (d, *J *= 4 Hz, 1H), 4.96 (s, 1H), 4.68 (s, 1H), 4.40 (t, *J *= 4 Hz, 1H), 4.25 (d, *J *= 4 Hz, 1H), 4.12 (d, *J *= 8 Hz, 1H), 3.99 (t, *J *= 4 Hz, 1H), 3.52 (d, *J *= 8 Hz, 1H), 3.38 (d, *J *= 12 Hz, 1H), 1.88 (q, *J *= 8 Hz, 2H), 1.01 (t, *J *= 8 Hz, 3H). ^13^C NMR (100 MHz, CDCl_3_) *δ* = 173.76, 168.95, 168.44, 157.54, 152.44, 150.12, 149.19, 146.80, 146.04, 140.89, 134.79, 131.20, 130.80, 129.08, 128.77, 128.64, 128.61, 128.59, 128.54, 128.51, 128.43, 128.39, 128.31, 127.59, 126.95, 125.42, 118.84, 118.62, 98.17, 86.82, 82.35, 77.34, 72.74, 67.82, 67.44, 66.25, 65.31, 53.41, 52.84, 52.80, 50.57, 50.01, 49.96, 46.47, 31.59, 7.82. IR (KBr): *ν* (cm^−1^) = 3474, 3414, 3232, 3146, 1744, 1658, 1616, 1502, 1399, 1230, 1153, 636, 479.

## 4. Proliferation inhibition assay

The inhibition ratio using the HCPT conjugate **3** was evaluated with standard 3-(4,5-dimethylthiazol-2-yl)-2,5-diphenyltetrazolium bromide (MTT) assays after 48 or 72 h of drug treatment. In a panel of human tumour cell lines, including human colon carcinoma SW-480 cell, human hepatocellular carcinoma HepG2, human gastric carcinoma BGC-803 cell and PANC-1 cells, conjugate **3** exhibited high inhibition. HepG2, SW480, BGC803 and PANC-1 cells were cultured in RPMI 1640 or McCoy's 5A medium (Invitrogen), supplemented with 10% heat-inactivated fetal bovine serum and 1% penicillin/streptomycin (Thermo Fisher Scientific). All cell lines were maintained at 37°C with 5% CO_2_. Cell viability was evaluated by the MTT assay.

A series of conjugated camptothecin norcantharidin 10-phenolic ester derivatives **3a–i** were designed and synthesized regio-selectively in a moderate yield. As shown in table 1, most of the camptothecin norcantharidin 10-phenolic ester compounds have similar activities against HepG2, BGC803, SW480 and PANC-1 cell lines *in vitro*, and these compounds will be further tested against different cell lines *in vivo*.

**Table 1.** *In vitro* antitumor activities (inhibition/%) of camptothecin analogues **3a–i**.^a^

	HepG2	BGC-803	SW480	PANC-1
solvent^b^	1.16	0.93	1.02	1.04
cantharidin	78.08	75.33	75.04	77.21
camptothecin	74.19	73.95	71.04	73.88
compound **3a**	64.70	67.76	55.54	65.83
compound **3b**	68.94	70.19	64.91	67.49
compound **3c**	63.08	68.49	58.09	68.41
compound **3d**	69.04	68.88	60.82	68.99
compound **3e**	68.11	60.65	61.91	67.07
compound **3f**	66.37	56.19	67.16	68.77
compound **3g**	73.68	72.81	70.61	71.87
compound **3h**	68.79	70.44	69.31	68.71
compound **3i**	73.85	55.79	62.02	70.56

^a^Preliminary testing concentration *c* = 50 µM.

^b^Test solvent DMSO.

## 5. Results and discussion

**Scheme 3.**
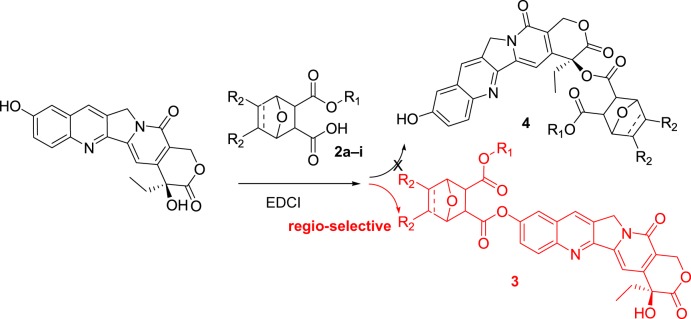
The direct coupling of HCPT with norcantharidin analogues.

HCPT and norcantharidin are both commercially available materials, which were thus chosen as starting materials to prepare this target molecule (**I**) [19]. In our recent published literature [20], a sealed tube promoted coupling of camptothecin with norcantharidin can easily produce 20(*S*)-*O*-linked ester in a moderate-to-high yield. Continued with this finding, the coupling of HCPT with norcantharidin derivatives was also expected to form the corresponding 20(*S*)-*O*-linked ester (**4**). However, to our surprise, a 10-*O*-linked phenolic ester was formed in the presence of EDCI and DMAP, DCM as a solvent under reflux condition (scheme 3).

**Figure 3.**
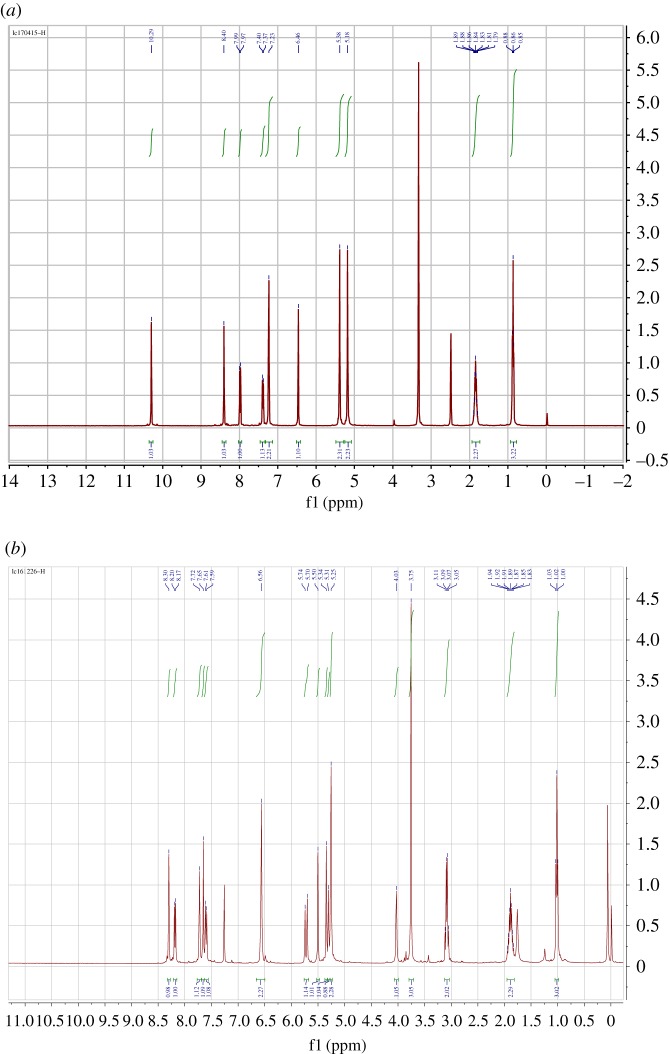
^1^H NMR spectra of HCPT (*a*) and conjugate **3a** (*b*).

To determine which of the OH groups participated in the coupling reaction of HCPT with norcantharidin, we measured ^1^HNMR spectrum of HCPT using DMSO-d_6_ as solvent. It is not difficult to find that the phenolic hydroxyl group lies in *δ* = 10.22 ppm and alcoholic hydroxyl group lies in *δ* = 6.4 ppm. Thus, in ^1^H NMR spectrum of product **3a** prepared from the coupling of HCPT and norcantharidin monomethyl ester **2a**, the peak of 10-phenolic hydroxyl group disappeared completely, which strongly indicates that the phenolic hydroxyl group participates in the coupling reaction (figure 3).

Thus, norcantharidin monoacid monoesters **2a–i** were chosen as the materials to couple with HCPT in the condition of EDCI and DMAP at ambient temperature for 2 days to give the corresponding product. The results are summarized in table 2.

**Table 2.** Direct coupling of HCPT with norcantharidin monoesters **2a–i**. 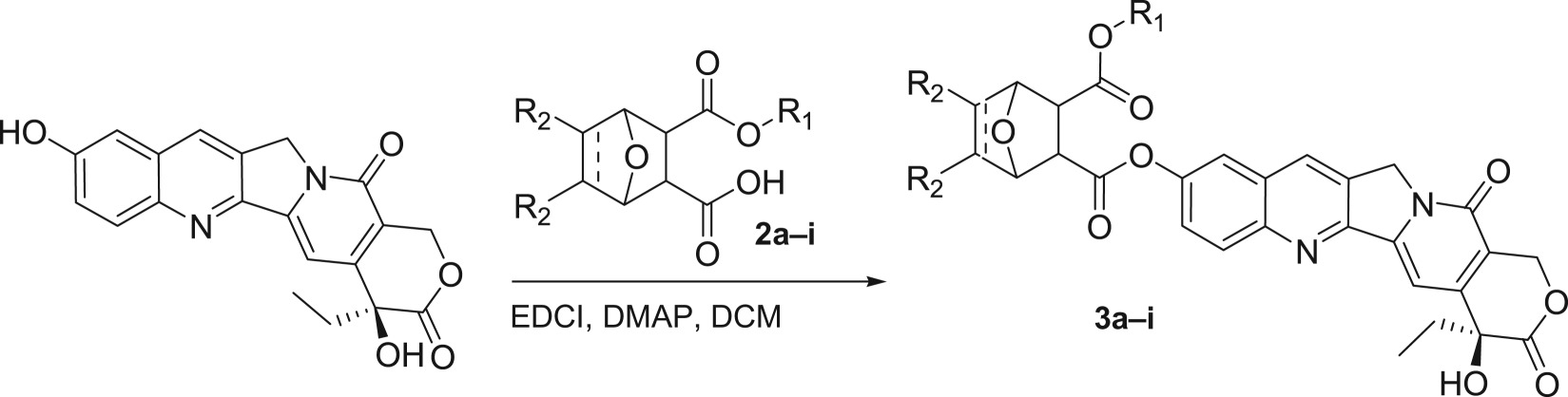

substrate	*R*_1_	*R*_2_	coupling reagent	solvent	temp.	time	yield (%)
HCPT + **2a**	CH_3_	H	EDCI/DMAP	DCM	RT	2 d	69
HCPT + **2b**	C_2_H_5_	H	EDCI/DMAP	DCM	RT	2 d	65
HCPT + **2c**	Bn	H	EDCI/DMAP	DCM	RT	3 d	44
HCPT + **2d**	CH_3_	H	EDCI/DMAP	DCM	RT	2 d	67.3
HCPT + **2e**	Et	H	EDCI/DMAP	DCM	RT	2 d	66.3
HCPT + **2f**	Bn	H	EDCI/DMAP	DCM	RT	2 d	44.6
HCPT + **2g**	CH_3_	Br	EDCI/DMAP	DCM	RT	2 d	62.2
HCPT + **2h**	Et	Br	EDCI/DMAP	DCM	RT	2 d	55.3
HCPT + **2i**	Bn	Br	EDCI/DMAP	DCM	RT	2 d	48

## 6. Conclusion

A series of HCPT norcantharidin 10-phenolic esters were selectively synthesized via the coupling of HCPT with norcantharidin mono acid esters. The synthesized target compounds were characterized by ^1^H NMR and ^13^C NMR. This series of compounds have also shown a strong activity against several cancer cell lines and synergistic effects were observed.

## Supplementary Material

NMR spectrum;IR spectrum

## Data accessibility

Data are available as part of the electronic supplementary material.
