# Supplementary material for: The regio-selective synthesis of 10-hydroxy camptothecin norcantharidin conjugates and their biological activity evaluation in vitro
Source: R Soc Open Sci. 2018 Jun 13;5(6):172317. doi: 10.1098/rsos.172317 (PMC6030274; doi:10.1098/rsos.172317)

**Compound 3a ：^1^H-NMR**

**
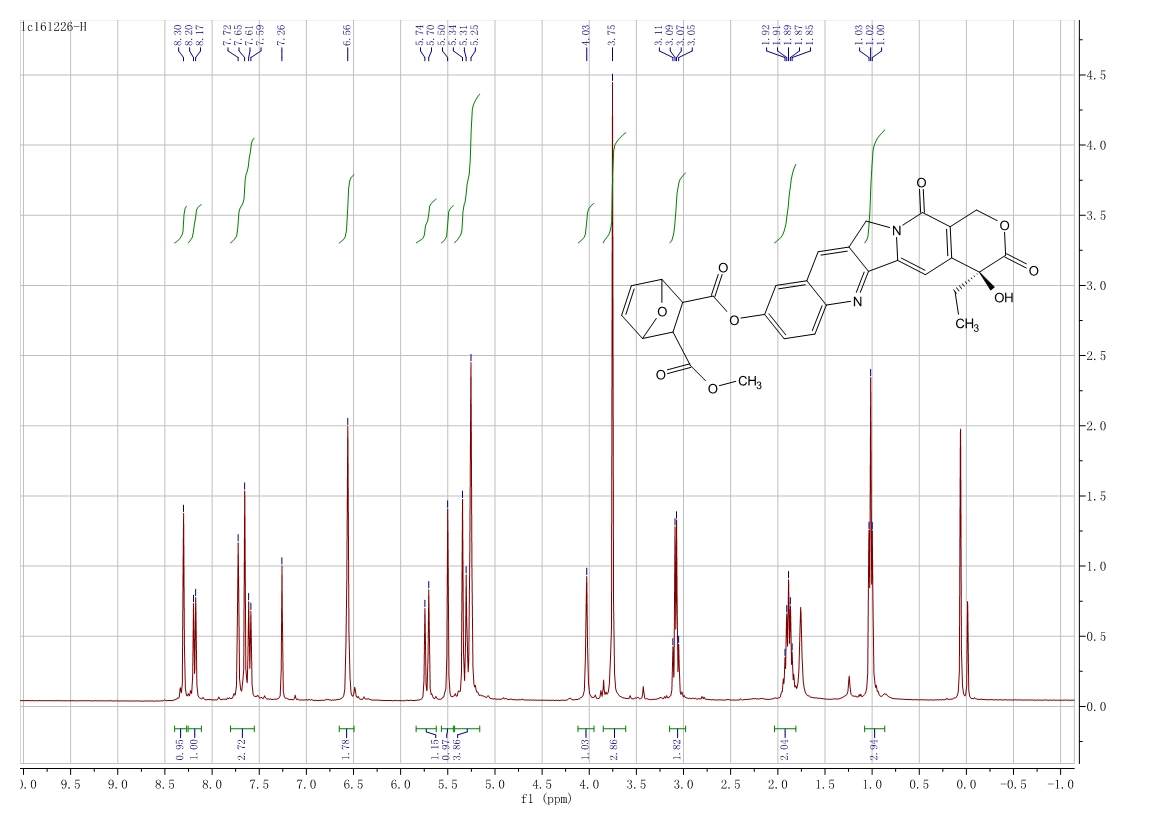
Compound 3a：^13^C-NMR**

**
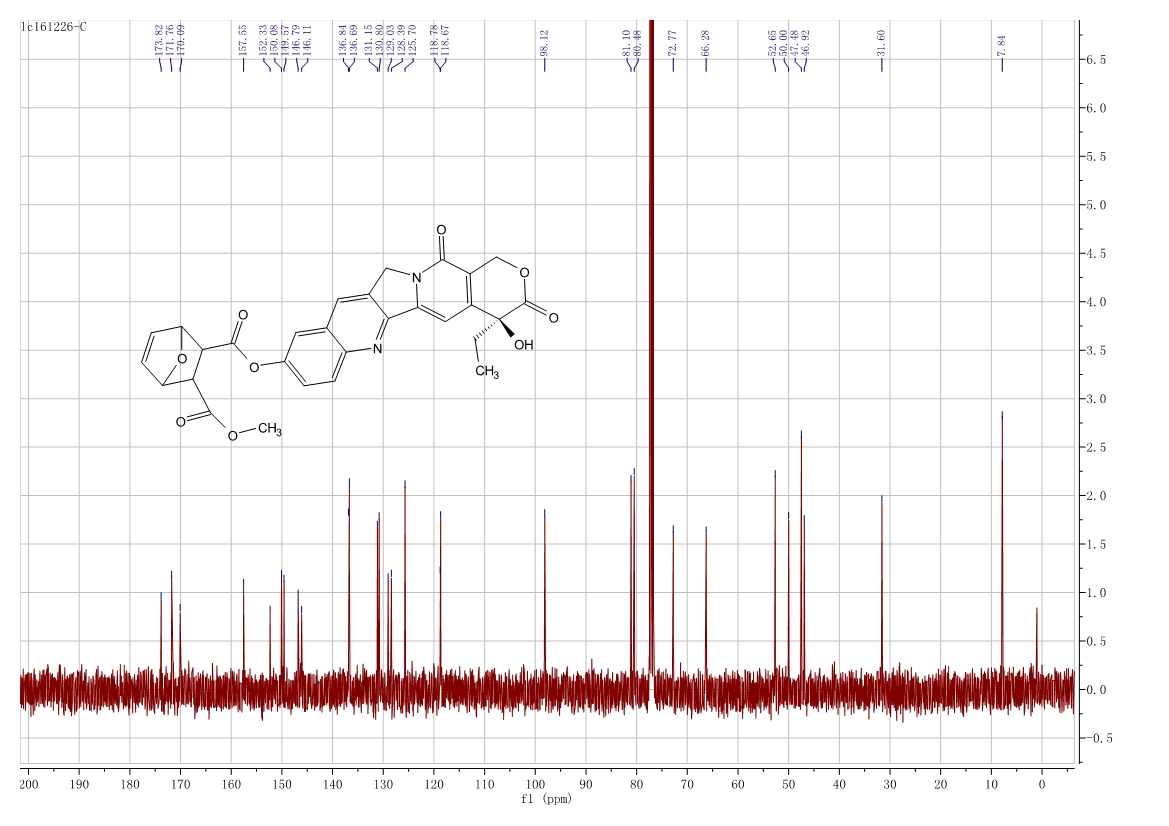
**

**Compound 3b：^1^H-NMR**

**
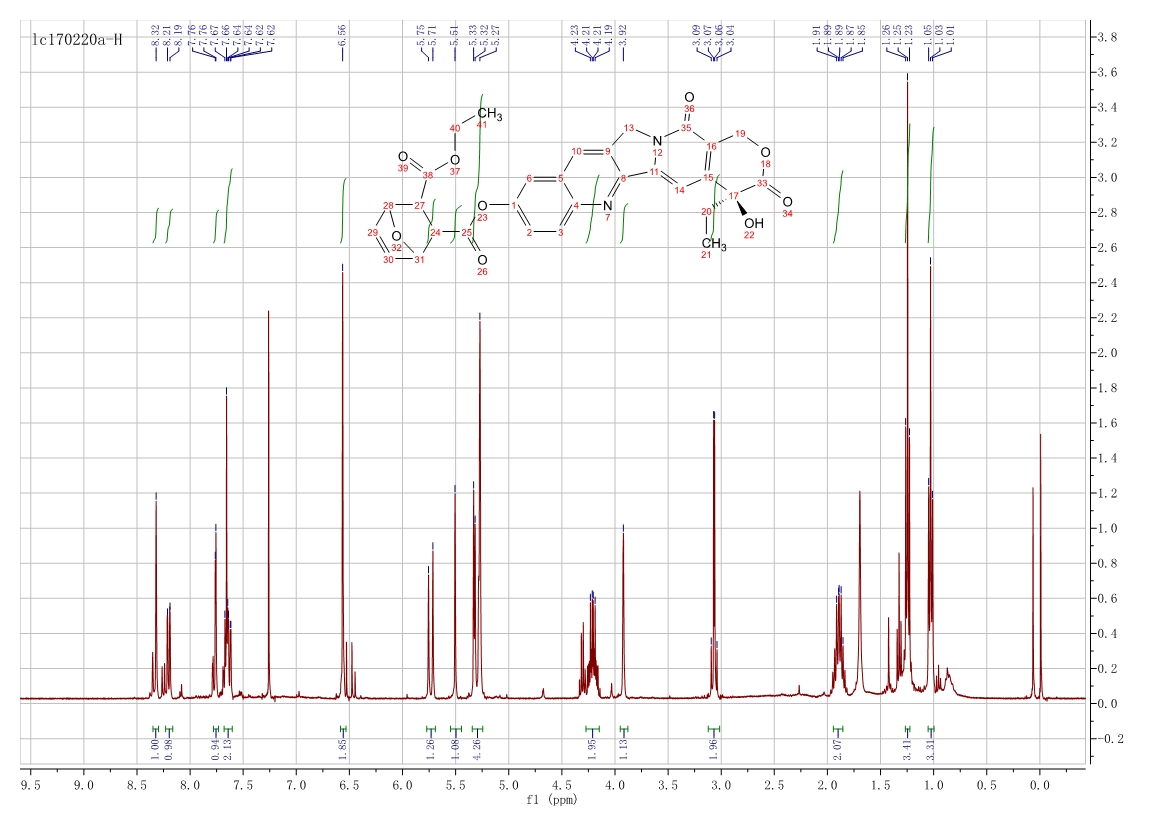
**

**Compound 3b：^13^C-NMR**

**
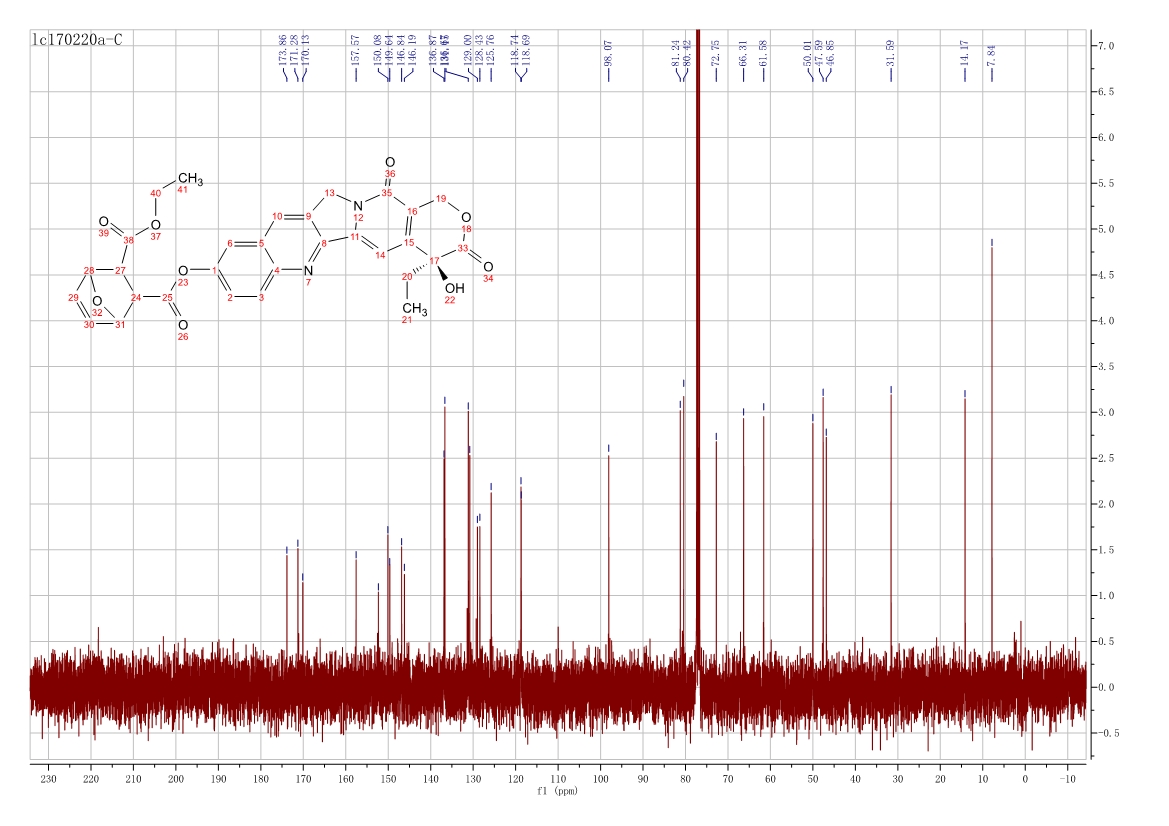
**

**Compound 3c：^1^H-NMR**

**
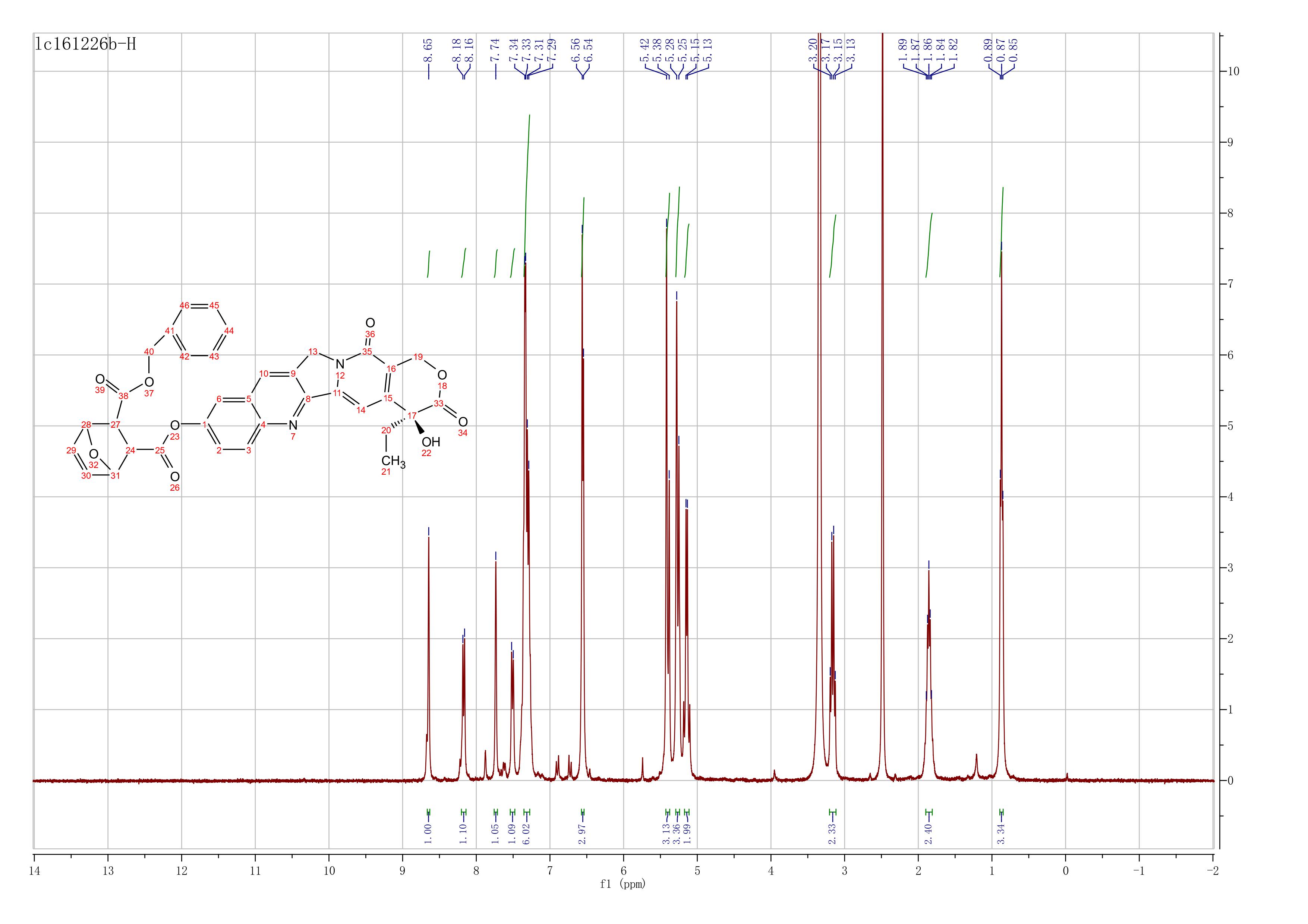
**

**Compound 3c：^13^C-NMR**

**
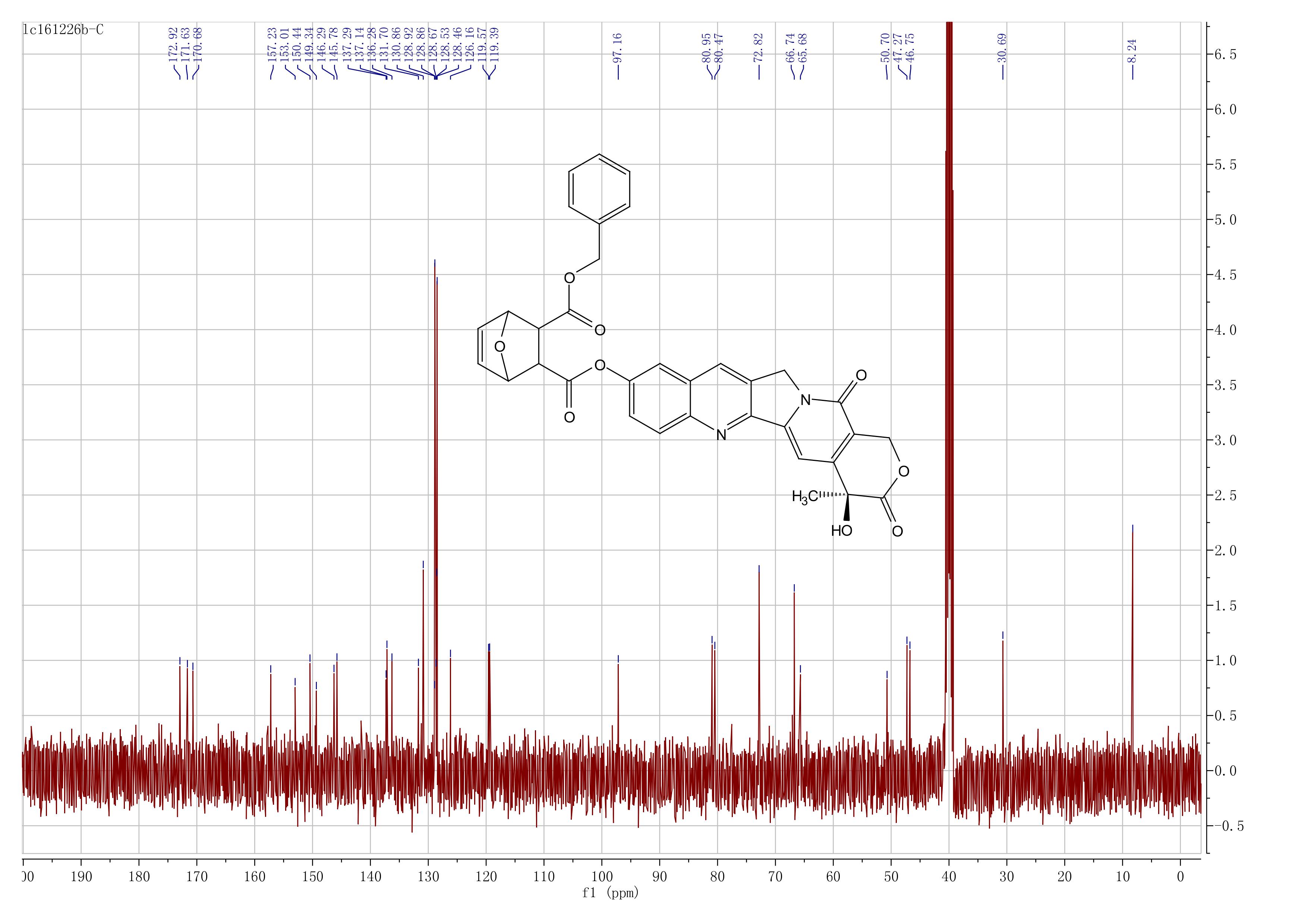
**

**Compound 3d：^1^H-NMR**

**
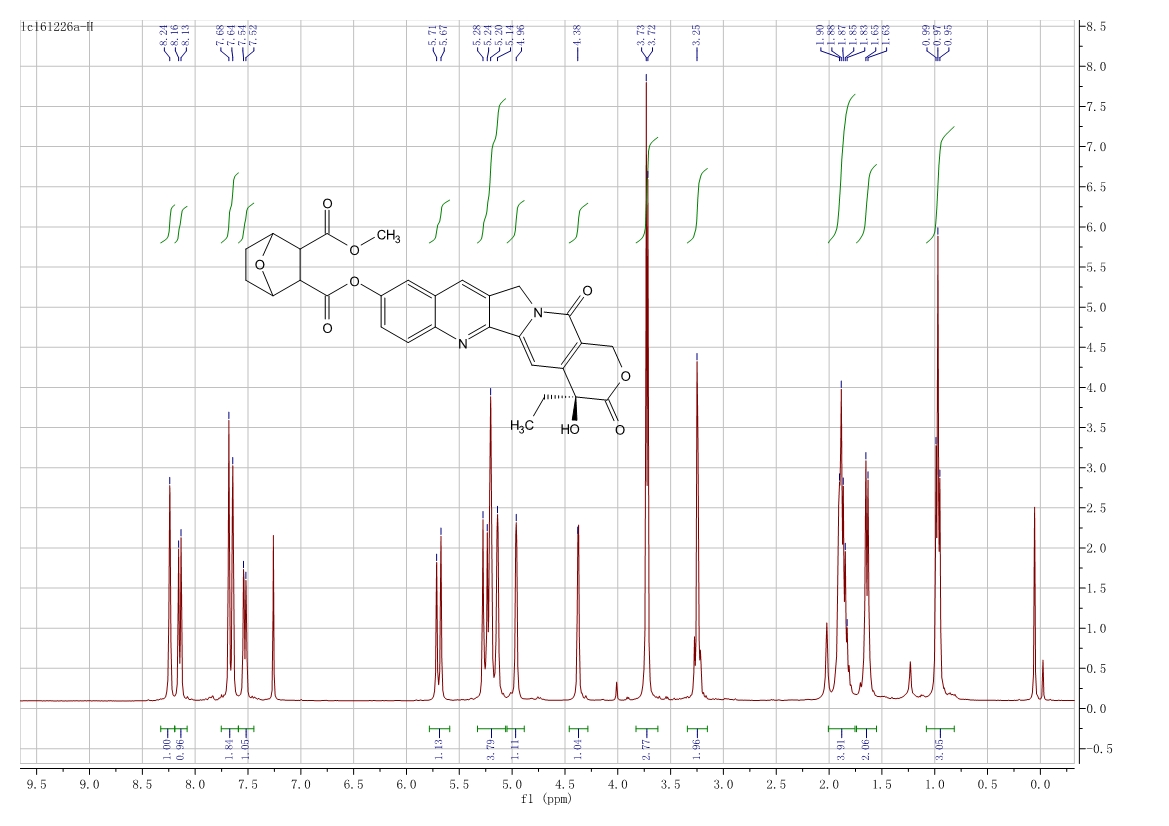
**

**Compound 3d：^13^C-NMR**

**
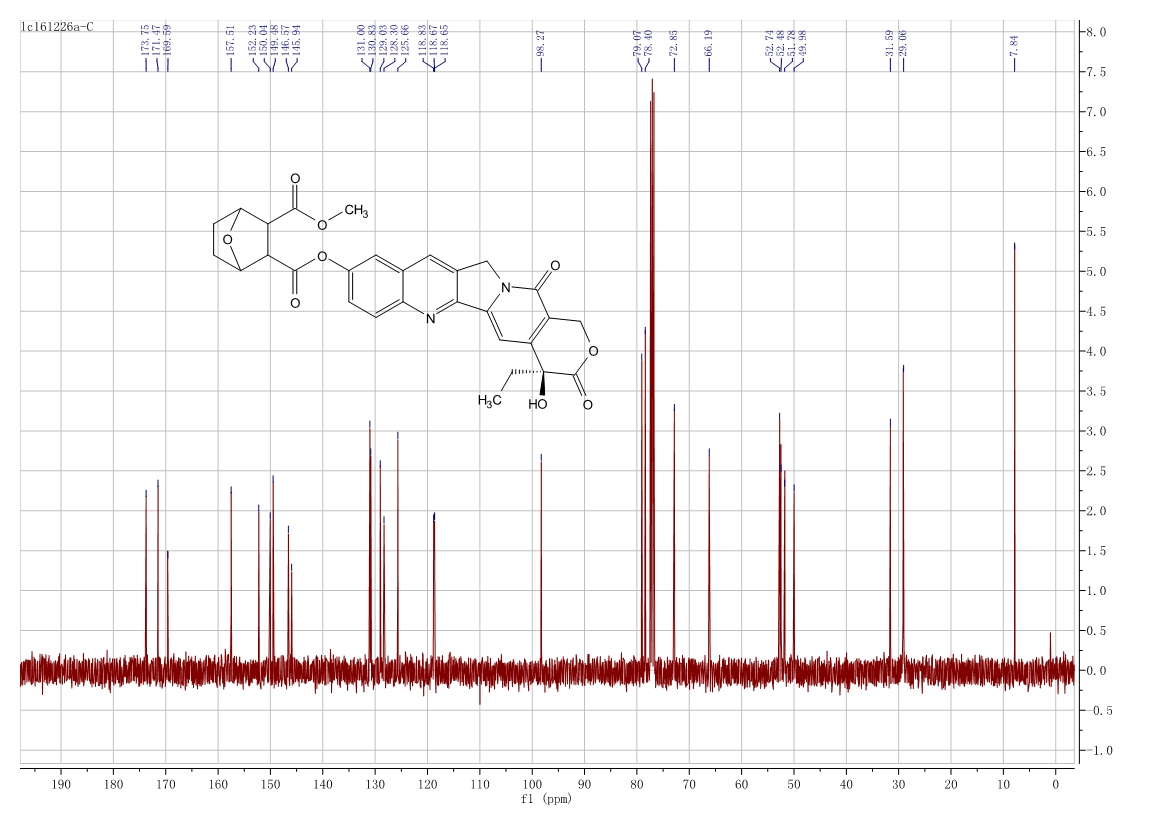
**

**Compound 3e：^1^H-NMR**

**
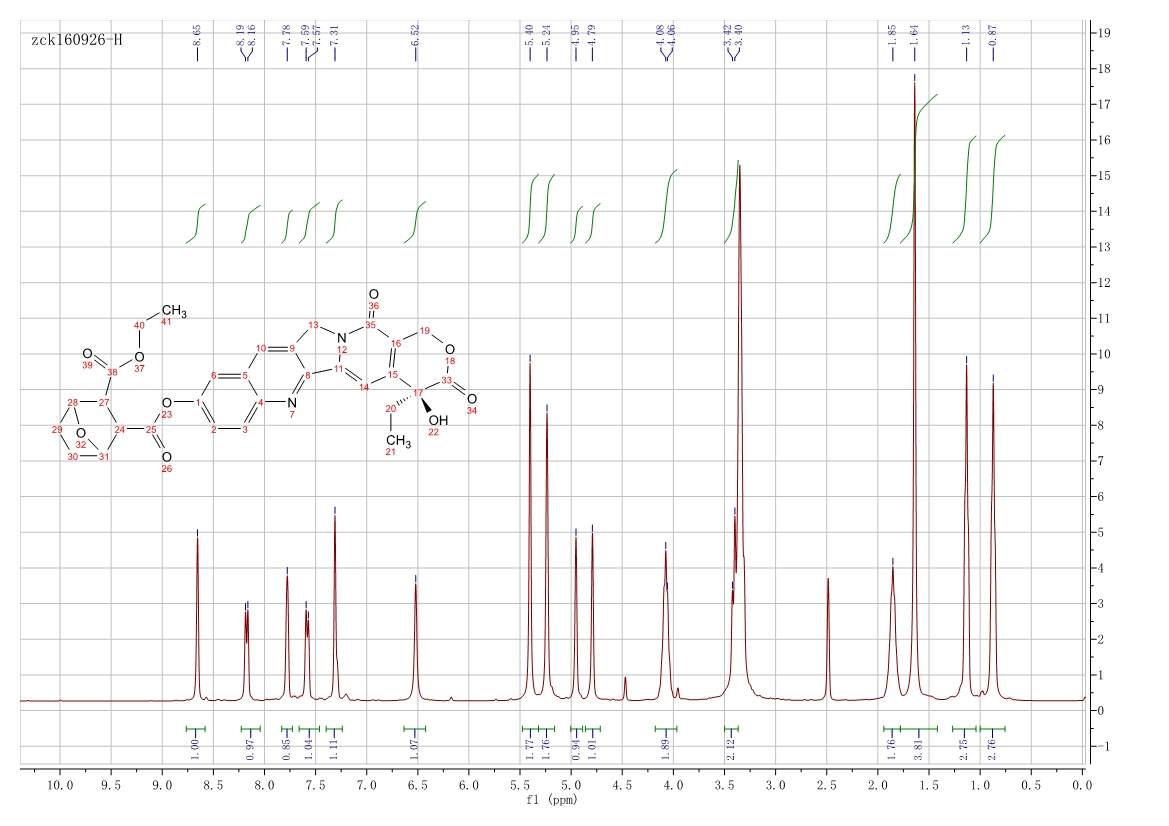
**

**Compound 3e：^13^C-NMR**

**
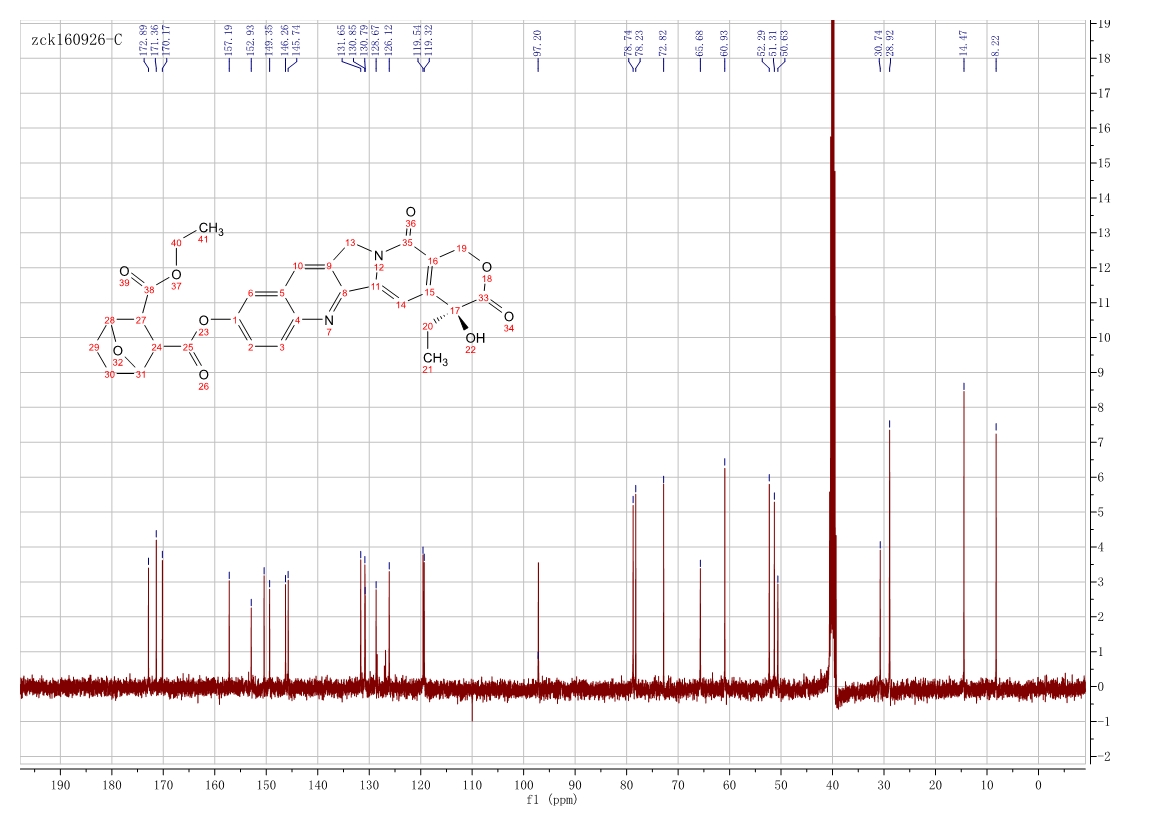
**

**Compound 3f：^1^H-NMR**

**
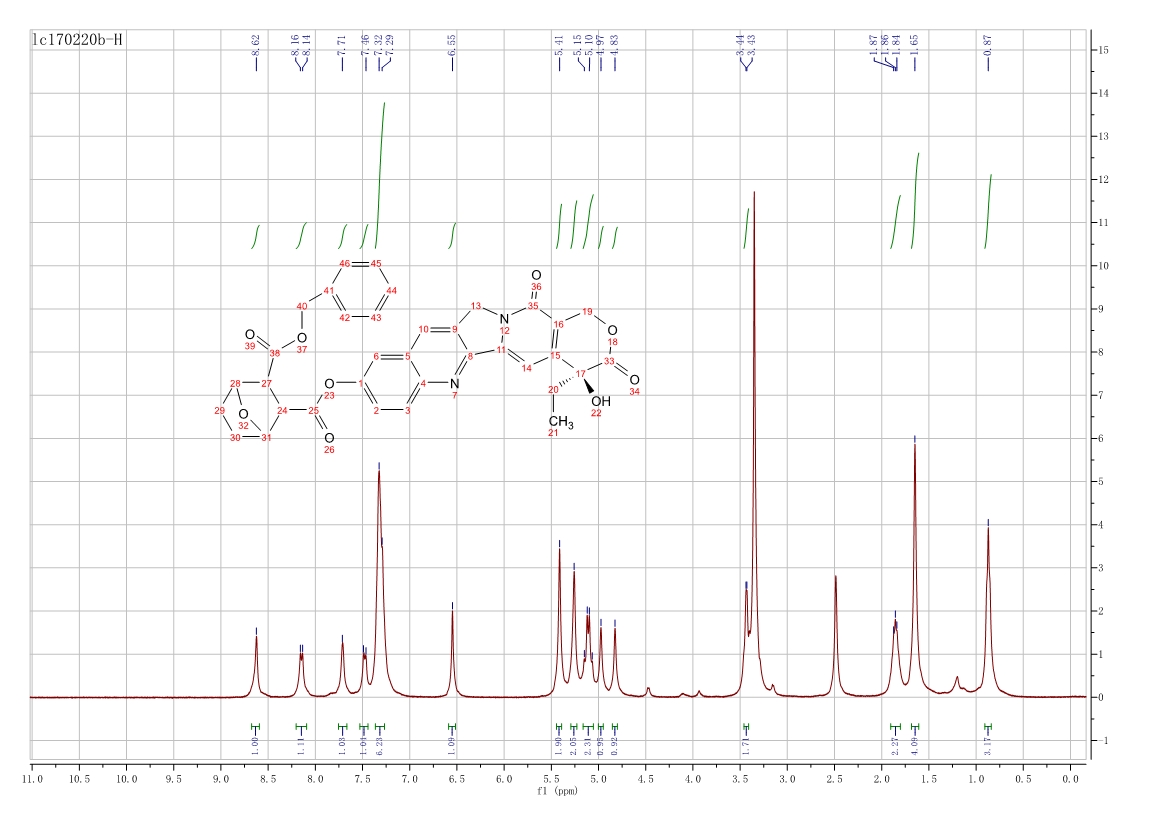
**

**Compound 3f：^13^C-NMR**

**
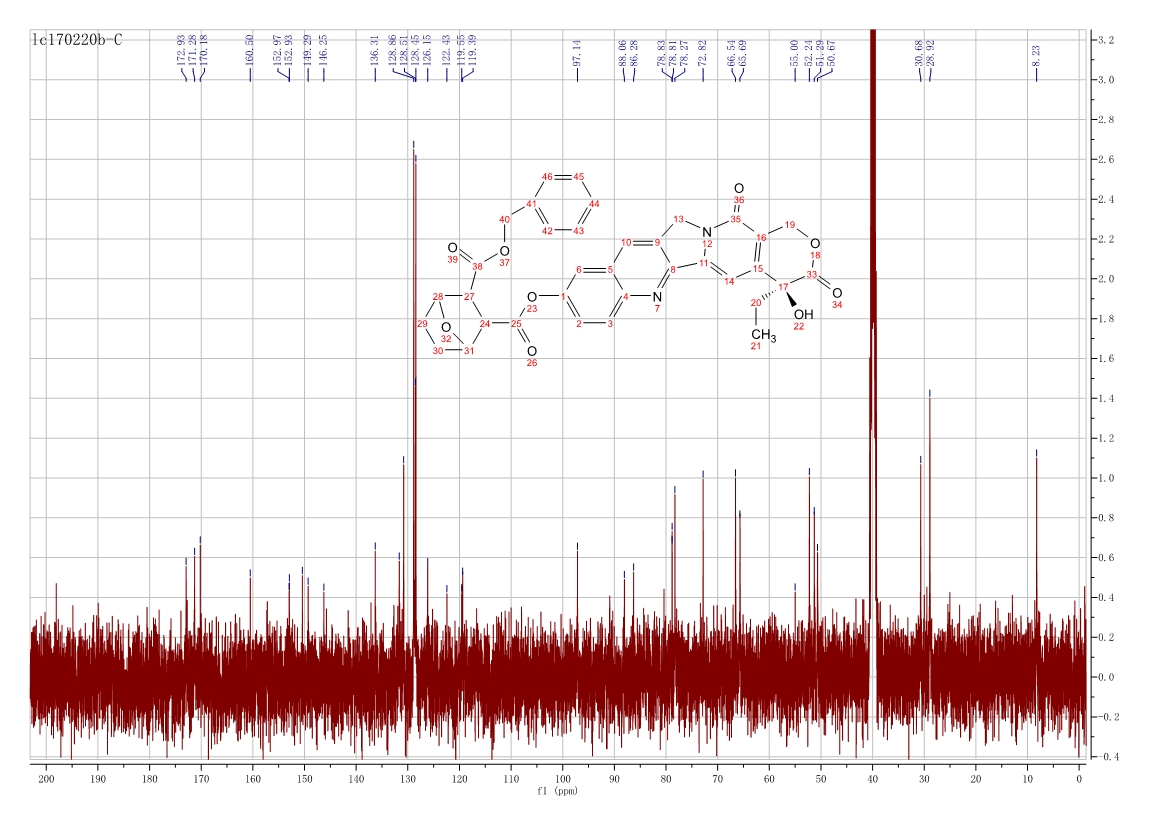
**

**Compound 3g：^1^H-NMR**

**
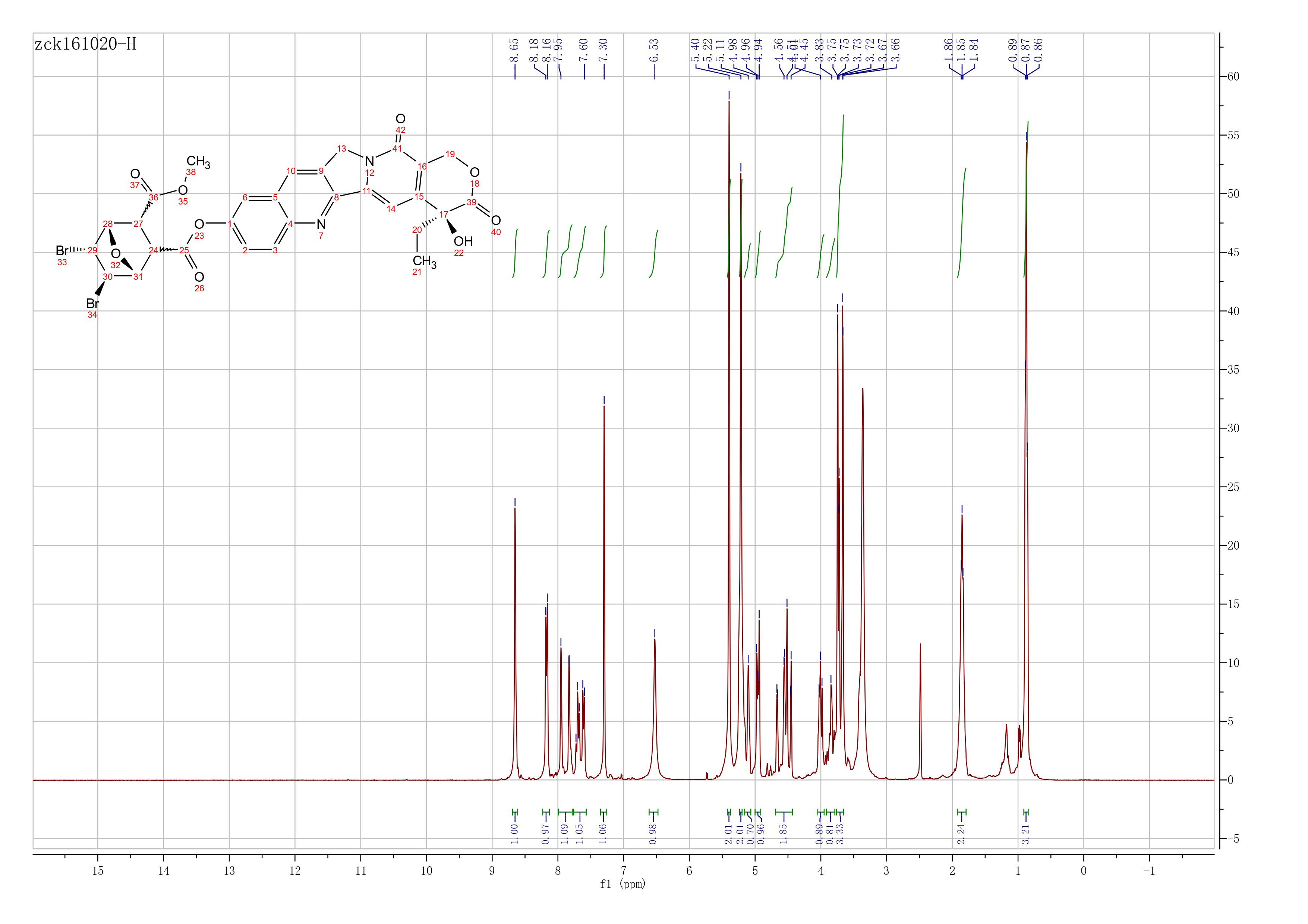
**

**Compound 3g：^13^C-NMR**

**
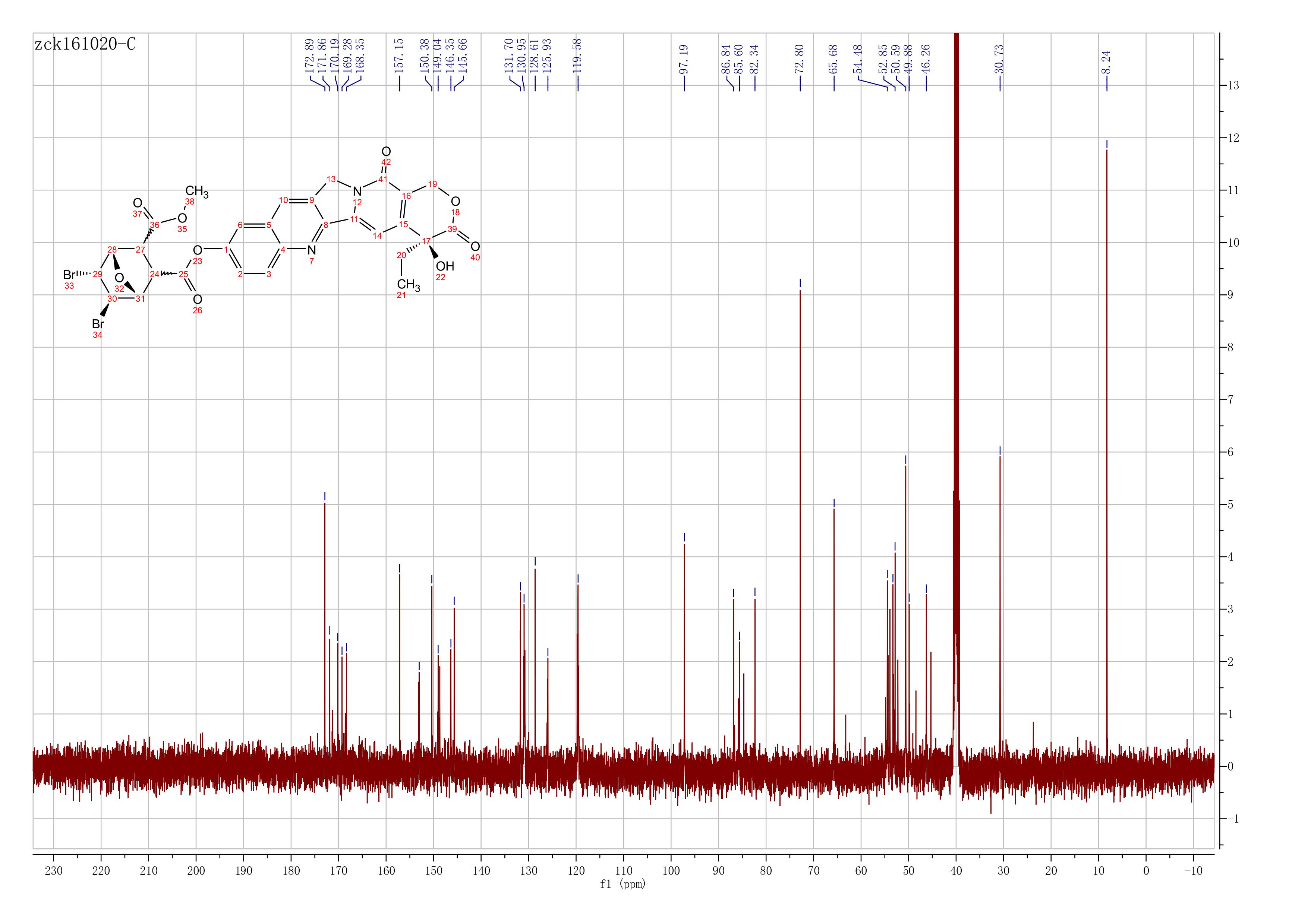
**

**Compound 3h：^1^H-NMR**

**
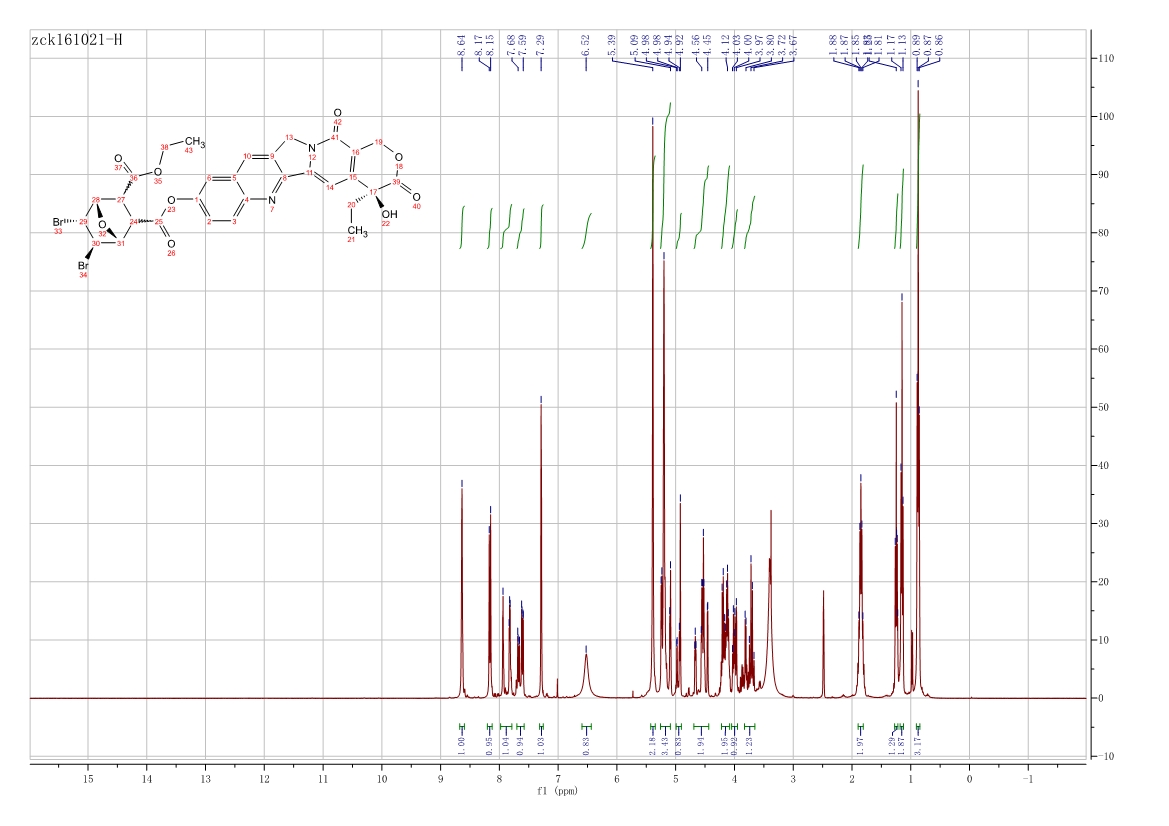
**

**Compound 3h：^13^C-NMR**

**
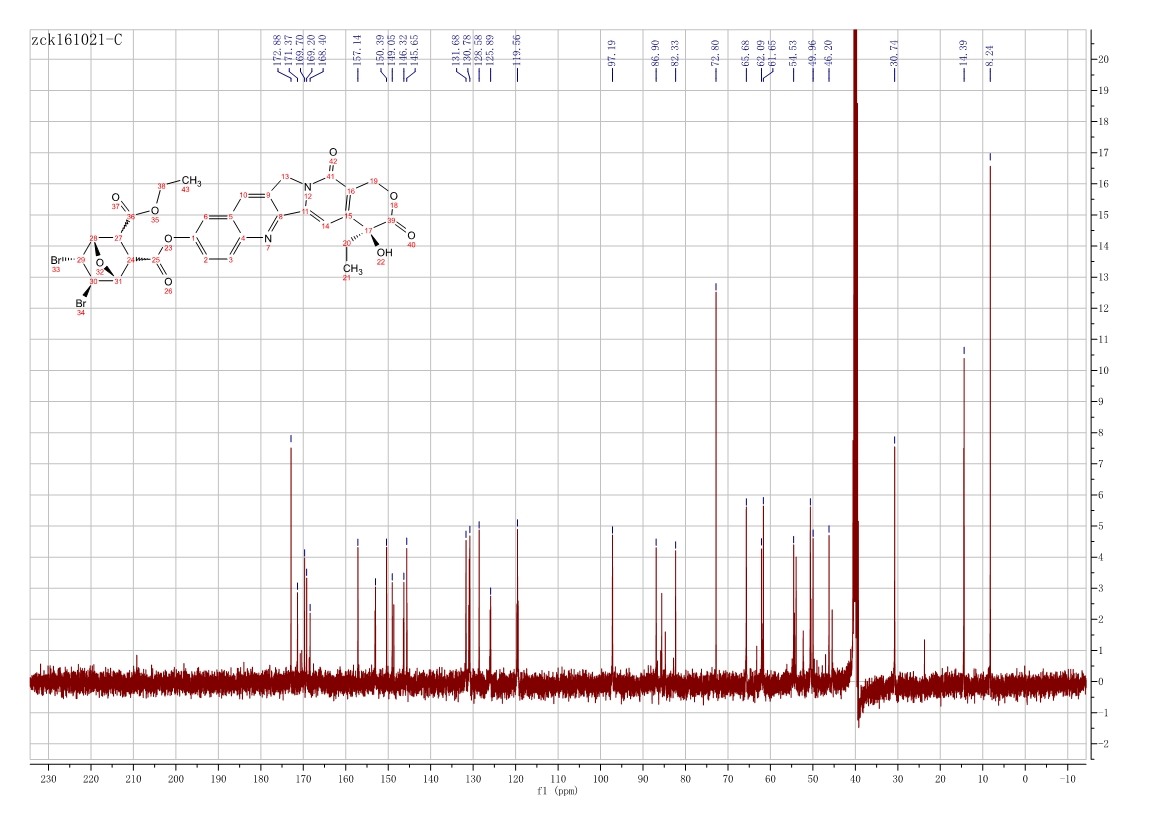
**

**Compound 3i：^1^H-NMR**

**Compound 3i：^13^C-NMR**

**Compound HCPT：^1^H-NMR**

**IR spectrum：**

**Compound 3a**


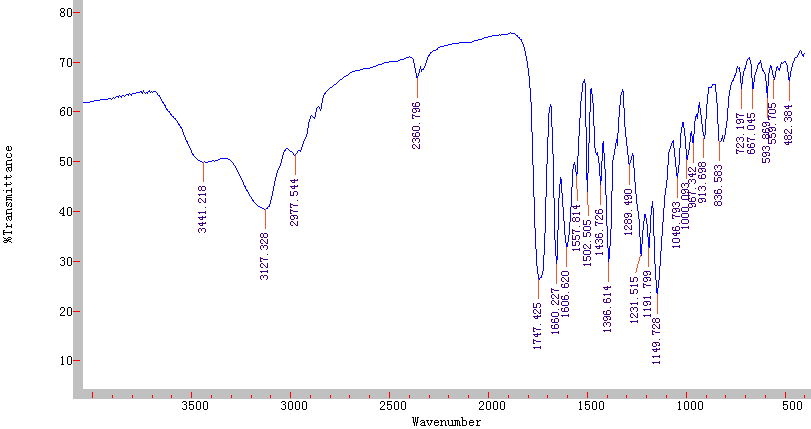


**Compound 3b**


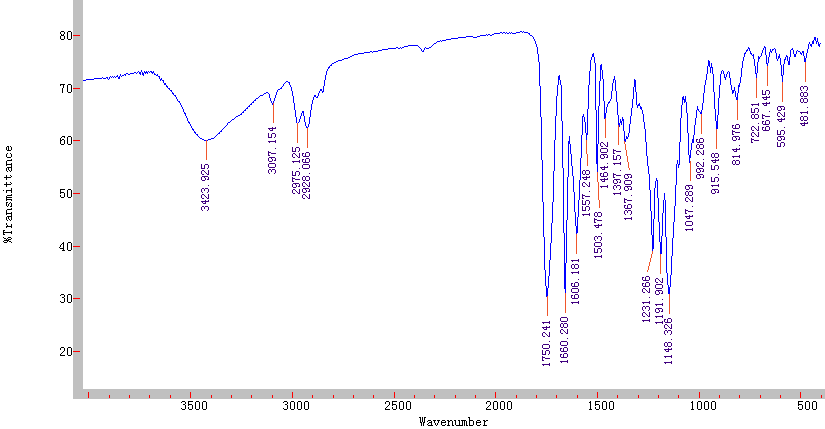


**Compound 3c**


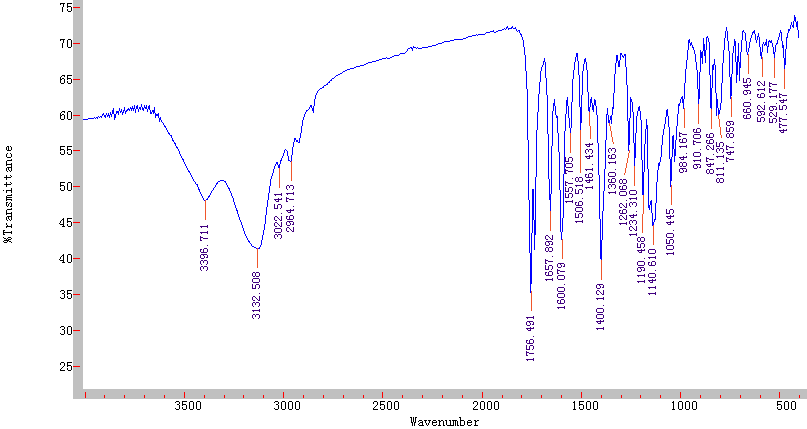


**Compound 3d**


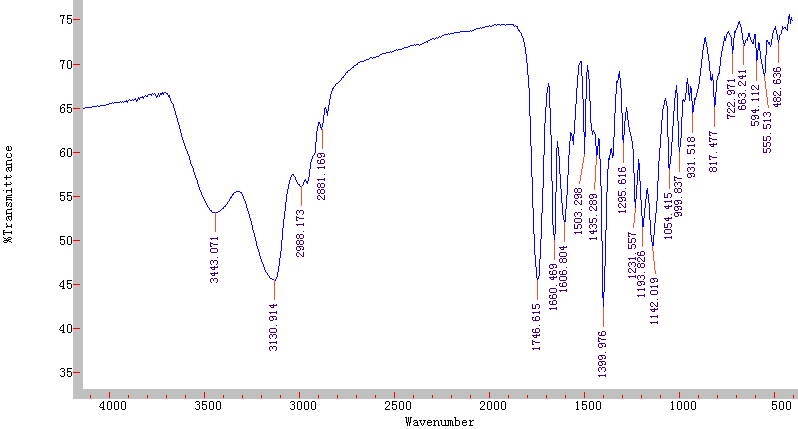


**Compound 3e**


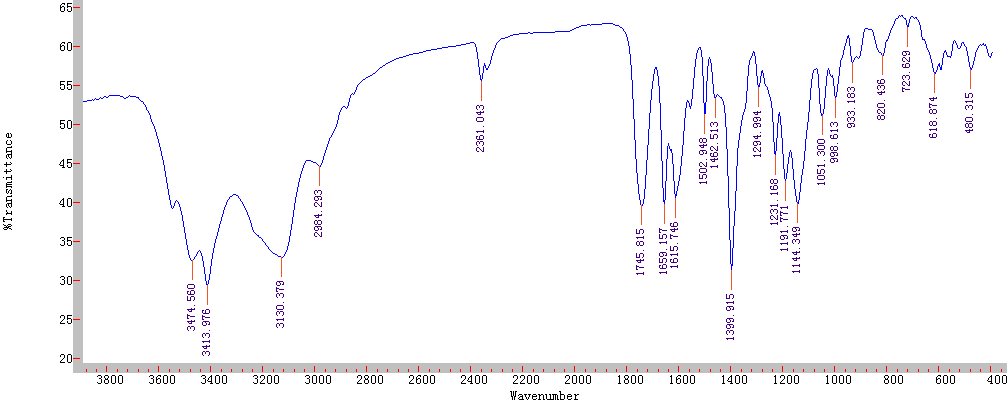


**Compound 3f**


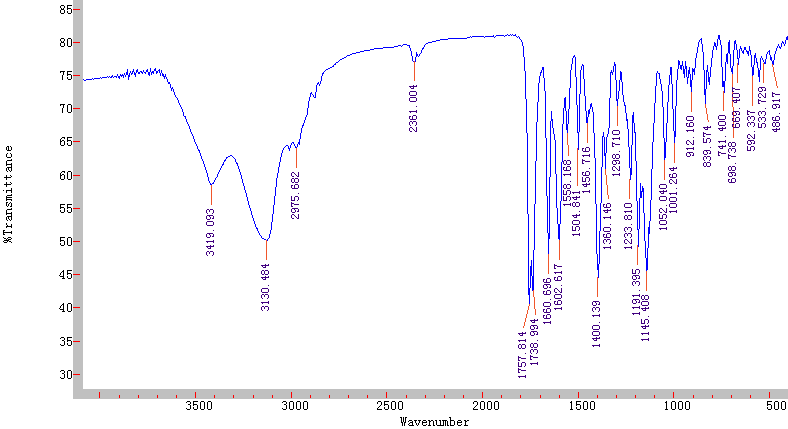


**Compound 3g**


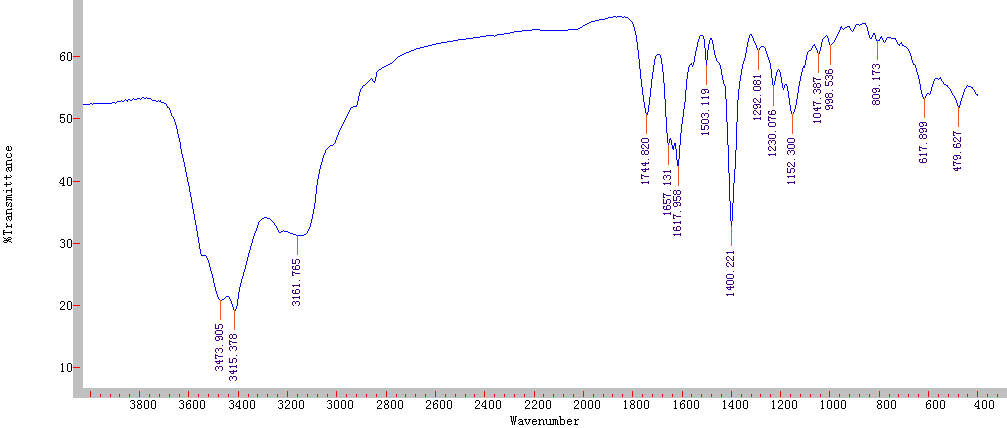


**Compound 3h**


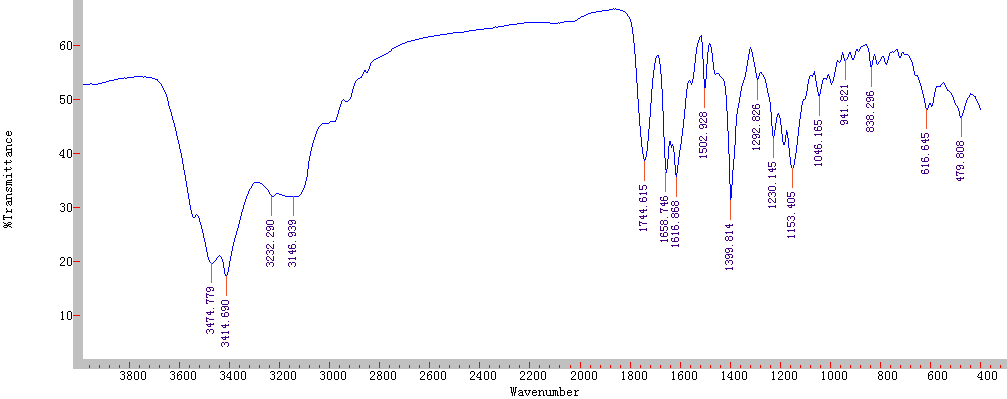

Supplement: NMR spectrum;IR spectrum [file rsos172317supp1.doc]
